# The Diels-Alder Cross-Linked Gelatin/Dextran Nanocomposite Hydrogels with Silver Nanoparticles for Wound Healing Applications: Synthesis, Characterization, and In Vitro Evaluation

**DOI:** 10.3390/gels10060408

**Published:** 2024-06-19

**Authors:** Iman Gholamali, Sung-Han Jo, Won Han, Juhee Lim, Ali Rizwan, Sang-Hyug Park, Kwon Taek Lim

**Affiliations:** 1 Industry 4.0 Convergence Bionics Engineering, Pukyong National University, Busan 48513, Republic of Korea; imangholamali1212@pknu.ac.kr (I.G.); josunghan91@gmail.com (S.-H.J.); hanwon0427@naver.com (W.H.); hee9752@naver.com (J.L.); 2 Department of Smart Green Technology Engineering, Pukyong National University, Busan 48513, Republic of Korea; arizwan92@outlook.com; 3 Major of Biomedical Engineering, Division of Smart Healthcare, College of Information Technology and Convergence, Pukyong National University, Busan 48513, Republic of Korea; 4 Institute of Display Semiconductor Technology, Pukyong National University, Busan 48513, Republic of Korea

**Keywords:** Diels-Alder cross-linked, hydrogels, silver nanoparticles, wound healing

## Abstract

Wound healing involves a sophisticated biological process that relies on ideal conditions to advance through various stages of repair. Modern wound dressings are designed to imitate the natural surroundings around cells and offer properties such as moisture regulation, strength, and antimicrobial defense to boost healing. A recent research project unveiled a new type of gelatin (Gel)/dextran (Dex) hydrogels, linked through Diels-Alder (D-A) reactions, loaded with silver nanoparticles (Ag-NPs) for cutting-edge wound treatment. Gel and Dex were chemically modified to form the hydrogels via the D-A reaction. The hydrogels were enriched with Ag-NPs at varying levels. Thorough analyses of the hydrogels using methods like NMR, FT-IR, and SEM were carried out to assess their structure and nanoparticle integration. Rheological tests displayed that the hydrogels had favorable mechanical attributes, particularly when Ag-NPs were included. The hydrogels demonstrated controlled swelling, responsiveness to pH changes, and were non-toxic. Testing against E. coli showcased the strong antibacterial activity of the nanocomposite hydrogels in a concentration-dependent manner. This investigation showcased the promise of these bioactive nanocomposite hydrogels in promoting speedy wound healing by maintaining a moist environment, offering an antimicrobial shield, and ensuring mechanical support at the wound site.

## 1. Introduction

In recent years, the sciences related to biomaterials have advanced tremendously. These developments have mostly aimed to come up with groundbreaking answers to many hard medical issues, especially those connected to helping wounds heal. Injuries or sores that do not heal well or at all put a huge strain on both those providing medical care and people dealing with such conditions, making it extremely important to design versatile materials that can actively improve how tissue repairs itself and fight associated complications [1,2,3]. The sequence of steps involved in wound recovery is very complex, involving many biological substances and cell activities working together. Effective treatment depends on having materials available that help wounds stay moist enough to heal but also actively propel each phase of the healing process along [4,5].

Modern wound care requires materials that can support the complex wound healing process. Hydrogels are mostly water-based polymer networks that can absorb large amounts of water without dissolving. This allows them to mimic natural tissue and maintain a moist environment to promote healing [6]. Traditional hydrogel dressings are limited as they lack key attributes such as mechanical strength, antimicrobial activity, and controlled drug release which promote repair. Researchers are now exploring composite hydrogels combining beneficial natural polymers and nanocellulose, graphene, or nanoparticles to improve mechanical and functional properties [7,8]. Collagen, the main protein in skin, plays a vital role in healing. Gelatin (Gel) derived from collagen is body-compatible and helps cells grow and the regeneration of skin tissue, promoting the faster healing of wounds. Incorporating Gel into hydrogels mimics skin’s natural environment, assisting cell infiltration critical to mending. These hydrogels can absorb exudates and maintain a moist environment, thereby protecting the wound and enhancing the healing process [9,10]. Dextran (Dex), a hydrophilic polysaccharide, is widely applied in biomedical applications due to its non-immunogenic, non-antigenic, and non-toxic properties. Adding Dex to hydrogels can boost strength, swelling behavior and degradation over time, enhancing the dressing’s effectiveness. It encourages wound hydration and helps absorb exudate from wounds thereby keeping the wound bed clean and moist [11,12]. Developing nanocomposite hydrogels shows promise for providing multiple helpful properties to treatments. Including materials such as Gel and Dex provides structural support and biochemical signals speeding recovery [13]. Silver nanoparticles (Ag-NPs) have drawn interest due to potent antimicrobial effects against harmful microbes including drug-resistant strains, preventing infections that delay healing. Also, Ag-NPs can help to reduce inflammation around the wound area. By mitigating inflammation, they promote a more favorable environment for tissue repair and regeneration [14,15]. Embedding Ag-NPs into these nanocomposite hydrogels shows promise for providing needed antimicrobial properties to allow sustained silver ion release protecting wounds continuously from pathogens, fostering sterile repair. Ag-NPs demonstrate broad-spectrum efficacy and are widely researched for their controlled release and distinct physical and chemical properties in wound healing [16,17,18]. When choosing a polymer to stabilize silver nanoparticles (Ag-NPs), a key factor is its ability to form empty spaces within its polymeric structure during swelling. These voids will act as nucleation sites for nanoparticle formation and growth. For use as a wound dressing, the polymer is often formulated as a hydrogel using natural, synthetic, or semi-synthetic materials. Polymers from natural sources are most widely employed due to their biodegradability and compatibility with human tissue. The hydrogel structure allows the dressing to be easily applied to a wound site, where the silver nanoparticles can then release and take effect. Overall, the polymer network and hydrogel delivery system aim to support controlled silver ion release for optimal wound healing and antimicrobial activity [16].

The Diels-Alder reaction (D-A), a highly effective method in click chemistry, facilitates the creation of dynamic covalent cross-links within hydrogel networks. These cross-linkages not only bolster the mechanical robustness and architectural stability of hydrogels but also enable the precise release of therapeutic substances [19,20]. In the D-A reaction, a diene (a compound with two double bonds) engages with a dienophile (a molecule deficient in electron density that readily reacts with a diene) to generate a novel six-membered ring structure [21,22]. This procedure is remarkably efficient and extensively utilized in organic synthesis for fabricating intricate ring systems in a single step, establishing it as a preferred technique in the synthesis of pharmaceuticals, agrochemicals, and materials. D-A reactions stand out for their gentle reaction conditions and high yields, rendering them indispensable tools for chemists in various applications [23,24].

**Scheme 1:**
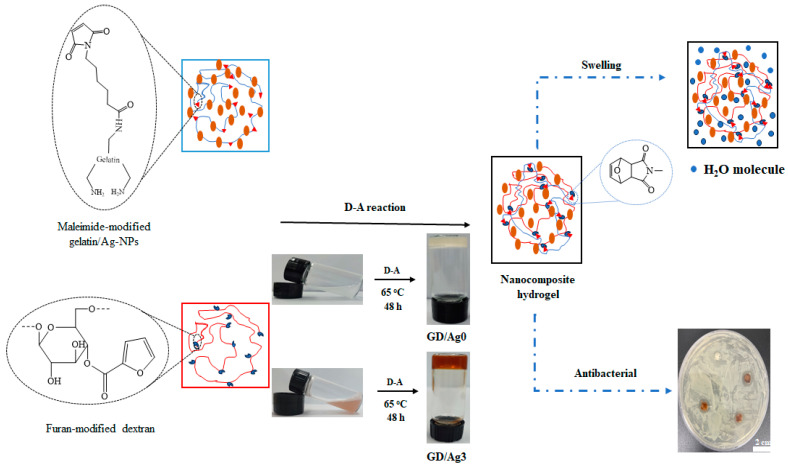
Schematic illustration of the preparation and properties of nanocomposite hydrogels.

In this research, we used the D-A reaction to craft a Gel/Dex hydrogel tailored for effective wound healing, as depicted in Scheme 1. The D-A reaction, a well-known technique in “click” chemistry, was crucial in creating precise chemically cross-linked hydrogels. Our method involved modifying Dex (to Dex-FA) with 2-furoic acid to kickstart the conjugated diene segment, while functionalizing Gel (to Gel-MI) with maleimide groups as dienophile segments. Gelation was performed by mixing Gel-MI containing AgNO_3_ with Dex-FA. Meanwhile, Gel acted as a mild reductant to reduce AgNO_3_ to Ag-NPs dispersed in the hydrogel matrix [16,25]. To comprehensively analyze the resulting hydrogel, we employed various analytical techniques, such as NMR, FE-SEM, EDS, FT-IR, XRD, and rheology tests. Furthermore, our study delved into pH-responsive behavior and the cell viability of the optimized hydrogel, confirming its effectiveness as a versatile solution for wound healing applications.

## 2. Results and Discussion

### 2.1. Preparation of Gel-MI and Dex-FA

**Scheme 2:**
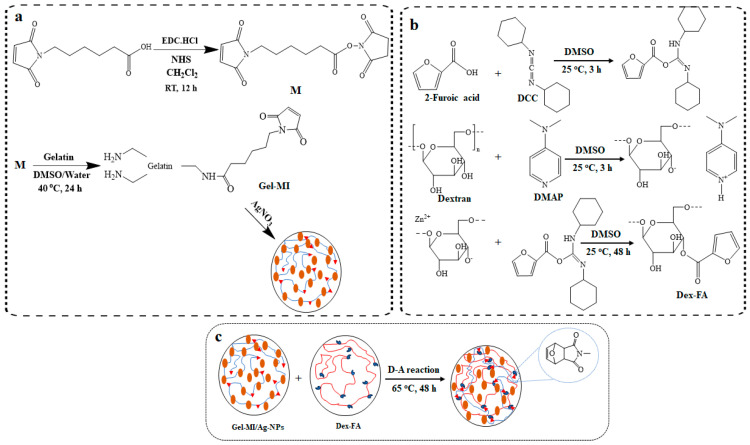
The reaction schematics of (**a**) preparation of Gel-MI; (**b**) Dex-FA.; (**c**) fabrication of nanocomposite hydrogels via D-A reaction.

**Figure 1:**
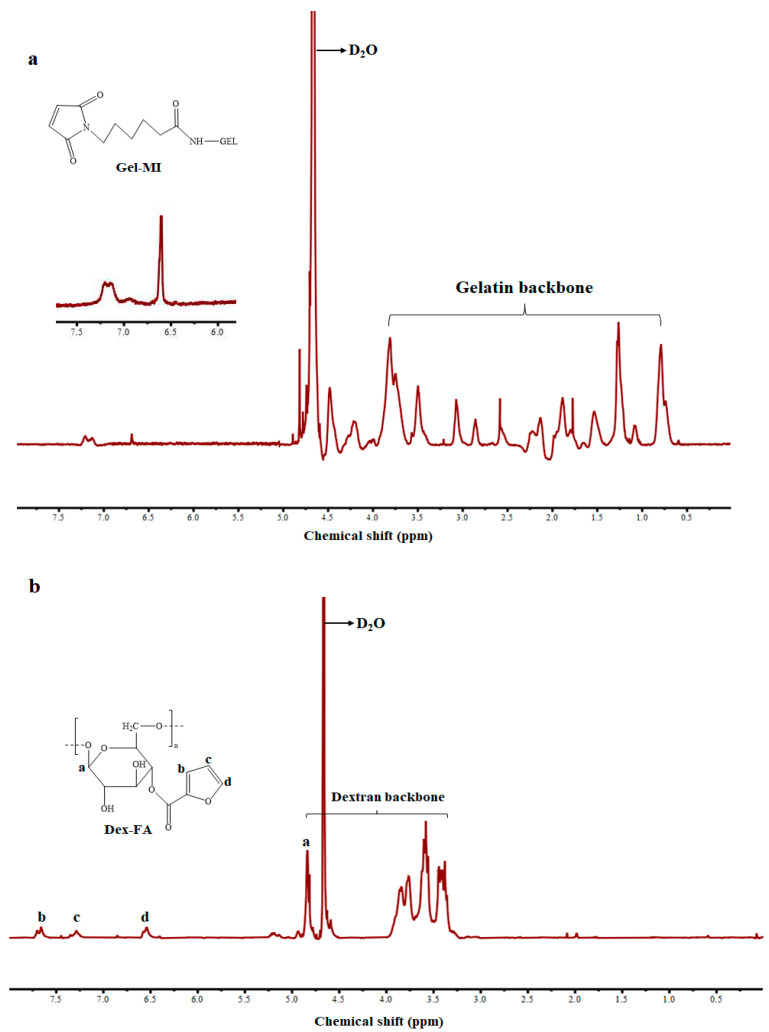
(**a**) ^1^H NMR spectrum of Gel-MI in D_2_O, (**b**) ^1^H NMR spectrum of Dex-FA in D_2_O.

The Gel-MI was created by linking 6-maleimidohexanoic acid with the Gel backbone using EDC/NHS as coupling agents. Similarly, Dex-FA was formed by attaching 2-furoic acid to the Dex backbone with DCC/DMAP as coupling agents. The synthetic pathways of Gel-MI and Dex-FA are shown in Scheme 2. The primary amines at the Gel backbone can act as a nucleophile, and therefore, can react with the COOH functional group of 6-maleimidohexanoic acid [26,27]. In order to prepare Gel-MI, we opted for a two-step bioconjugation process (Scheme 2a). Firstly, in situ NH active esters were prepared from 6-maleimidohexanoic acid, and the residues of EDC and NHS were removed by using a filter paper. The esters were further reacted with a Gel solution (pH ≈ 8–9) to obtain Gel-MI [28,29]. The conjugation of Gel with MI occurred by an amide linkage formation between the amine and carboxylic acid groups [30,31]. For Dex-FA, the diene groups were attached to the Dex through the reaction with FA. The catalyst DMAP, acting as a Lewis base, facilitated the polarization of hydroxyl groups on Dex, enabling the nucleophilic attack on the carbonyl ester of FA activated by DCC (Scheme 2b) [28]. The successful binding of maleimide to the Gel backbone was confirmed through ^1^H NMR and FT-IR analyses. The ^1^H NMR spectrum of the Gel-MI sample displayed signals at 2.86, 2.14, 1.26, and 1.08 ppm, representing the methylene protons of 6-maleimidohexanoic acid (see Figure 1a). Additionally, a singlet at 6.73 ppm indicated the presence of alkenyl protons in the maleimide moiety (Figure 1a). The quantity of the unreacted NH_2_ groups in Gel was measured by a ninhydrin assay and was used to calculate degree of substitution (DS), which was found to be 20.6% [10,31]. In the ^1^H NMR spectra of Dex-FA, the D_2_O signal was detected at 4.69 ppm, while peaks between 3.36 ppm and 5.19 ppm corresponded to the Dex backbone protons, with the anomeric proton at 4.84 ppm [32]. The α–1,4 linkages between glycosidic units were observed in the range of 4.82 ppm to 5.19 ppm, and signals at 6.54 ppm, 7.29 ppm, and 7.66 ppm confirmed the presence of FA protons, indicating successful derivatization. The DS of Dex was determined to be 9.3% (see Figure 1b) [28].

### 2.2. Hydrogel Formation through D-A Reaction

**Figure 2:**
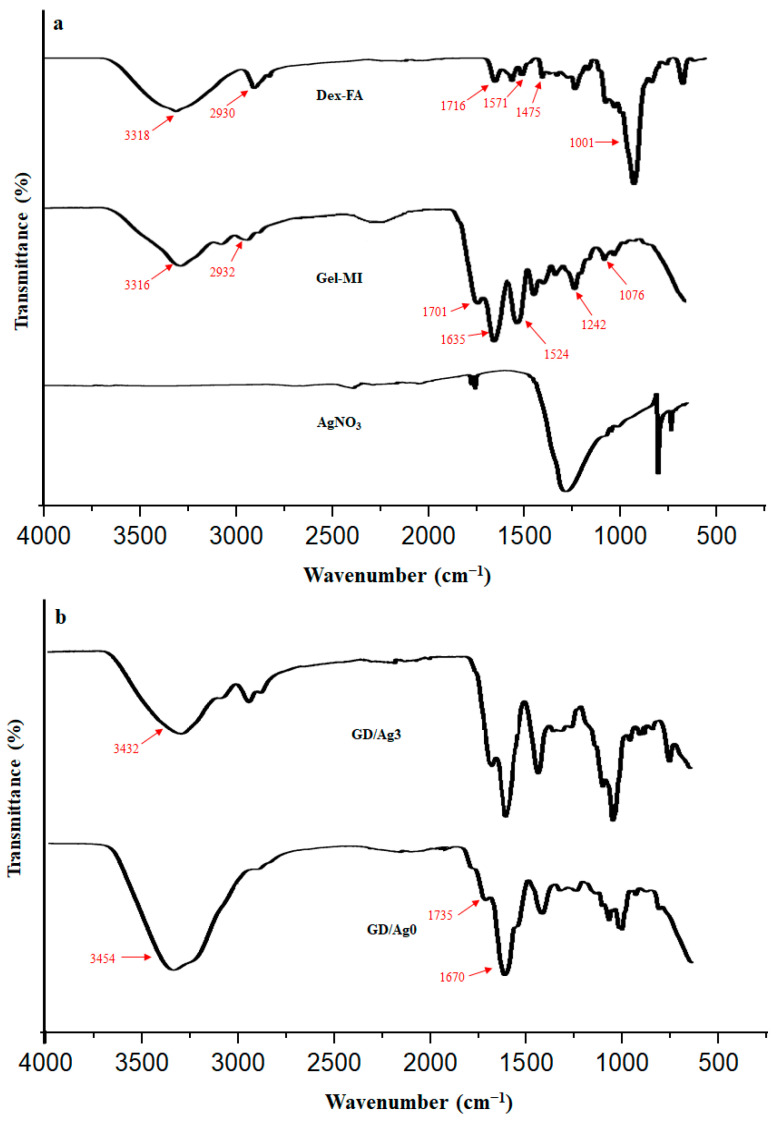
(**a**) FT-IR spectrums of AgNO_3_, Gel-MI, and Dex-FA. (**b**) FT-IR spectrum of GD/Ag0 and GD/Ag3.

Figure 2a illustrates the FT-IR spectra of Gel-MI, Dex-FA, and AgNO_3_. The FT-IR spectrum of Gel-MI shows a broad peak at 3316 cm^−1^ related to OH/NH_2_ stretching vibration. Additionally, peaks are observed at 2932 cm^−1^ for the C–H stretching vibration, 1635 cm^−1^ and 1524 cm^−1^ for amide I and amide II bands, and 1242 cm^−1^ and 1076 cm^−1^ for the C–O stretching vibration of C-OH [28]. The peak at 1701 cm^−1^ is attributed to the C=O stretching of the amide of maleimide, indicating the successful grafting of 6-maleimidohexanoic acid on Gel [30]. The FT-IR spectra of Dex-FA show peaks at different wavenumbers such as 3318 cm^−1^ (O–H stretching vibration), 2930 cm^−1^ (CH stretching vibrations), and 1001 cm^−1^ due to the carbon atoms of the α-1,6-linkages between the glucopyranose rings [33]. The furan-modified Dex spectrum presents peaks at 1475 cm^−1^ and 1571 cm^−1^ representing the skeletal stretching vibration of the C=C and at 1716 cm^−1^ due to the carbonyl stretch C=O ester in the furan ring. The presence of these peaks suggests a possible reaction between OH of the Dex molecule and the carbonyl group of 2-furoic acid [31]. The FT-IR spectra of AgNO_3_ contain an absorption peak at 500–800 cm^−1^ assigned to Ag stretching that confirms the existence of Ag-NPs in the polymer network. The chemical structure of the hydrogels was analyzed by FT-IR spectroscopy, as shown in Figure 2b. The bands corresponding to the maleimide ring’s carbonyl groups appear at 1735 and 1670 cm^−1^, and bands related to the stretching vibration of the C-O-C bond and C=C bonds in the D-A adduct are observed at 1450 and 1020 cm^−1^, confirming the cross-linking of the hydrogels by the D-A reaction [34,35]. In the FT-IR spectrum of the nanocomposite hydrogel, an O-H peak at lower wavelengths like 3432 cm^−1^ for GD/Ag3 compared to GD/Ag0 is observed due to intermolecular hydrogen bonding and the reaction of Ag-NPs with the Gel-MI/Dex-FA precursors.

**Figure 3:**
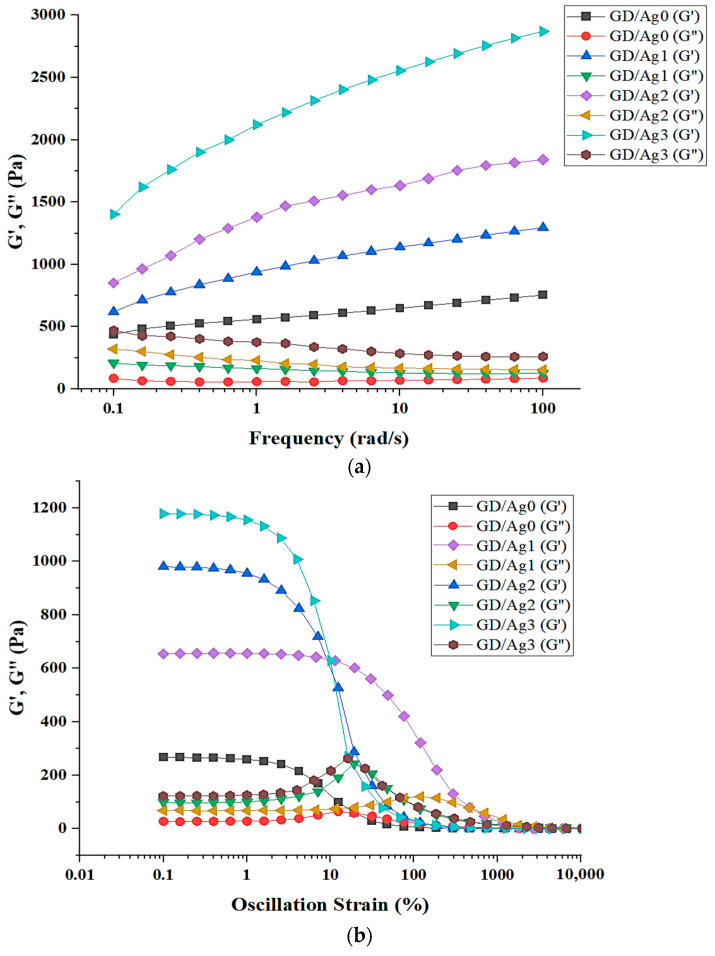
(**a**) The storage modulus (G′) and loss modulus (G″) of hydrogels as a function of angular frequency under 1% strain at 25 °C, (**b**) the strain amplitude sweep experiments.

Rheology provides important insights into how materials will behave under different conditions. For hydrogels intended for wound healing, it is particularly important to understand their rheological properties as these will determine how effectively they can deliver treatment at the wound site. Properties like elasticity, viscosity, and shear thinning influence a hydrogel’s performance [10]. Testing the storage and loss moduli (G′/G″) of hydrogels created from Gel/Dex can assess their mechanical strength across a range of conditions, as depicted in Figure 3a. Throughout the frequency sweep analysis, G′ consistently surpassed G″ in value, indicating the flexible nature of the hydrogels [28,36]. Despite this trend, initial measurements at the lowest frequency showed storage modulus values of 754 Pa for the hydrogel, and 1294, 1840, and 2869 Pa for the nanocomposite hydrogels are referred to as GD/Ag1, GD/Ag2, and GD/Ag3, respectively, as shown in Figure 3a. These findings highlight the direct correlation between mechanical strength and cross-linking density in hydrogels. This suggests that the elastic properties of hydrogels are more dominant than their viscosity characteristics, reflecting a stable three-dimensional lattice structure and robust mechanical strength [37,38]. The rheological study revealed that the storage moduli of the nanocomposite hydrogels exceeded those of the pure hydrogel across all frequency angles, indicating a more rigid network structure in the nanocomposite hydrogels compared to the pure hydrogel.

The study performed dynamic oscillatory strain amplitude sweep tests on hydrogels spanning a strain range of 0.1 to 10,000% at a constant angular frequency of 10 rad/s to investigate their viscoelastic properties. The goal was to pinpoint the linear viscoelastic region (LVR) of the hydrogels, showing that G′ and G′′ values within the LVR remained relatively stable despite the applied strain, indicating typical viscoelastic behavior. The nanocomposite hydrogels displayed LVRs ranging from 0.1 to 2.9%, 0.1 to 4.2, 0.1 to 0.7, and 0.1 to 1.1%, referred to as GD/Ag0, GD/Ag1, GD/Ag2, and GD/Ag3, respectively. Generally, the LVR shrinks as the hydrogel transitions into a more solid (gel) state, as illustrated in the existing literature [39,40]. The decline in the hydrogel’s loss moduli observed at a final frequency of 100 Hz, compared to an initial frequency of 0.1 Hz in the nanocomposite hydrogels, surpassed that of the pure hydrogel, indicating higher gel strength in the nanocomposite versions. This boosted strength is credited to the physical cross-linking present in the nanocomposite hydrogel networks, influenced by the inclusion of Ag-NPs. These physical cross-links strongly bind the polymer chains in the hydrogel network, forming a sturdy structure with outstanding gel strength [41,42] (Figure 3b). 

**Figure 4:**
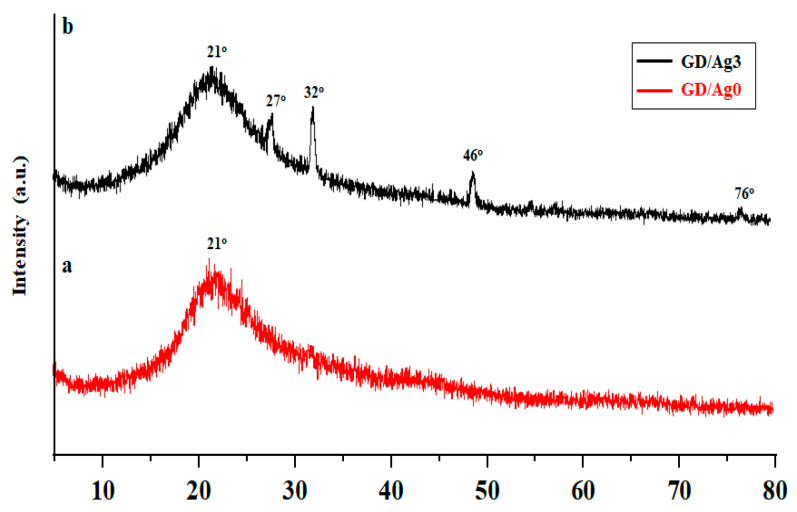
XRD patterns of (**a**) GD/Ag0 and (**b**) GD/Ag3.

Crystal structure analysis was conducted on the materials using X-ray powder diffraction (XRD). The materials tested included the Gel-Dex hydrogels and Gel-Dex/Ag-NP composite hydrogels. As shown in the Figure, the XRD patterns within the 2θ range of 5–80° provided information about the crystal structures present, which are illustrated in Figure 4 [38]. The diffractogram of the GD/Ag3 showed peaks around 27°, 32°, 46°, and 76° 2θ, demonstrating the inclusion of Ag-NPs within the Gel-Dex hydrogel network. No additional peaks were detected, validating the incorporation of Ag-NPs into the polymer matrix. A broad peak at 21° arose from the GD/Ag0 hydrogel polymer material itself. Therefore, XRD analysis identified the crystalline components in each material, confirming the production of Ag-NPs and embedded Ag-NPs within the different synthetic materials tested [16,43,44].

Ag-NPs exhibit distinctive and tunable optical properties due to their surface plasmon resonances, which are influenced by the nanoparticles’ shape and size distribution. Figure S1 presents the UV-Vis spectra of pure Gel/Dex hydrogels and Gel-Dex/Ag-NPs composite hydrogels. The nanocomposite hydrogels all display a broad absorption band in the UV range of 300–500 nm, attributed to the surface plasmon resonance effect. The intensity of the plasmon band increases with higher AgNO_3_ concentrations in the hydrogels, shifting towards higher wavelengths. This phenomenon is likely caused by an increase in the size of the Ag-NPs.

**Figure 5:**
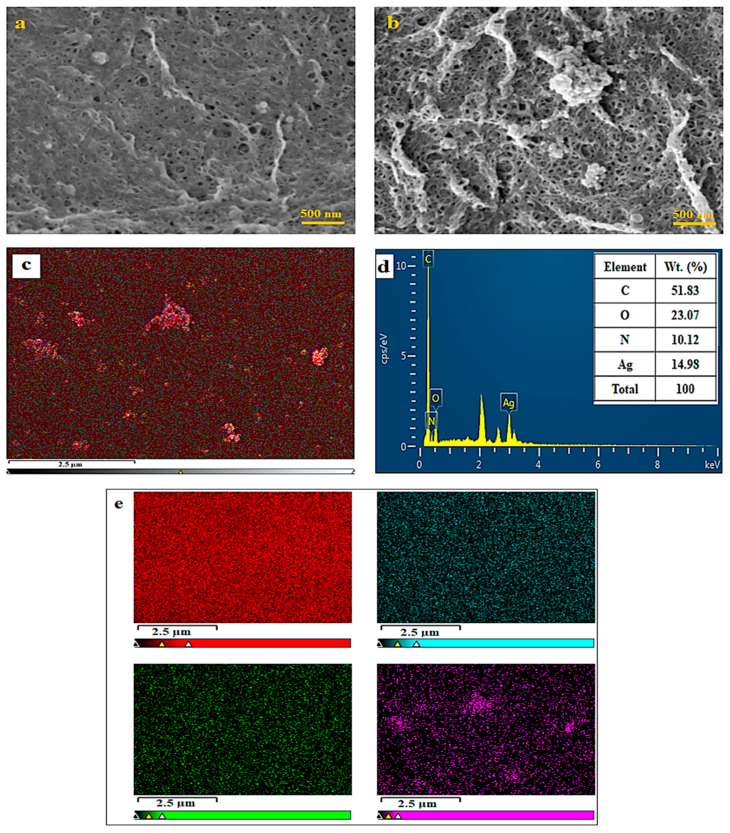
Morphological characterization of hydrogels. FE-SEM images of hydrogels (**a**) GD/Ag1 and (**b**) GD/Ag3. Scale bars showing 500 nm. The EDS of (**c**) the distribution map of Ag-NPs in the hydrogel structure and (**d**,**e**) the complete element distribution.

The freeze-dried nanocomposite hydrogel samples were examined under a magnification of 500 nm, as shown in Figure 5a,b. These images revealed that hydrogels synthesized through the D-A reaction exhibit an internal pore structure. The size, distribution, and connectivity of these pores play a crucial role in controlling the diffusion of bioactive molecules in various biomedical applications [28,45]. The analysis of Gel-Dex/Ag-NP hydrogels displayed a wrinkled surface with evenly spread pits. It is anticipated that the Gel-Dex/Ag-NP hydrogels would interact more with drug components due to their larger contact area, making them potentially effective for drug delivery and wound healing applications [44,46].

Figure 5c shows the EDS results of Gel-Dex/Ag-NP nanocomposite hydrogels. The analysis reveals the presence and distribution of Ag-NPs within the hydrogel structure, confirming its incorporation. Key elements identified on the surface of the nanocomposite hydrogel are C, O, N, and Ag, as depicted in Figure 5d,e. The EDS analysis suggests that the structure of the nanocomposite undergoes modification due to Ag-NPs. Moreover, it is likely that Ag-NPs play a role in altering the morphology and characteristics of the Gel-Dex/Ag-NP nanocomposite hydrogels [44]. 

### 2.3. Swelling Ratio Behavior

**Figure 6:**
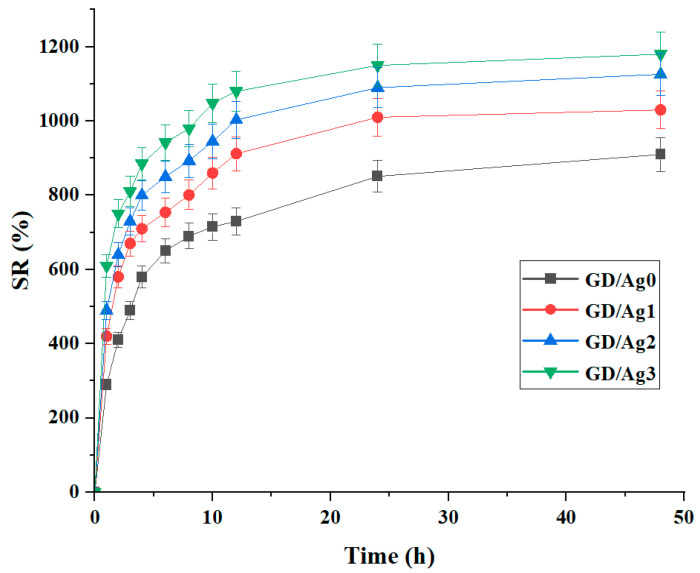
The swelling capability of nanocomposite hydrogels in PBS (pH 7.4).

In the realm of wound care, it is crucial for nanocomposite hydrogels to carefully regulate their swelling behavior. They should efficiently absorb tissue fluid effusion while avoiding excessive uptake of normal tissue fluid, which could impede wound healing. This underscores the necessity for pioneering hydrogels with reduced swelling to advance wound recovery [47]. The swelling characteristics of the hydrogels were analyzed within the physiological pH environment of the human body. In a pH 7.4 phosphate buffer, the swelling equilibrium for GD/Ag0 reached 910% within 48 h. Similarly, GD/Ag1, GD/Ag2, and GD/Ag3 exhibited swelling percentages of 1030%, 1070%, and 1180%, respectively, after the same incubation period, as depicted in Figure 6. The equilibrium swelling occurs when the osmotic pressure is balanced by the elastic retractive forces of the polymer chains. However, the presence of ionic groups in biopolymers and variations in pH induce alterations in osmotic pressure, leading to the establishment of a new swelling equilibrium. This results in electrostatic repulsion among the amine groups, fostering increased swelling. Furthermore, compared to GD/Ag0, nanocomposite hydrogels exhibit heightened swelling, possibly due to enhanced surface roughness, and the introduction of cavities from the added nanofillers. This increased swelling facilitates greater water penetration within the carrier network, offering potential benefits for wound healing [31,44,48,49].

### 2.4. Antibacterial Activity and In Vitro Cell Viability Assay

**Figure 7:**
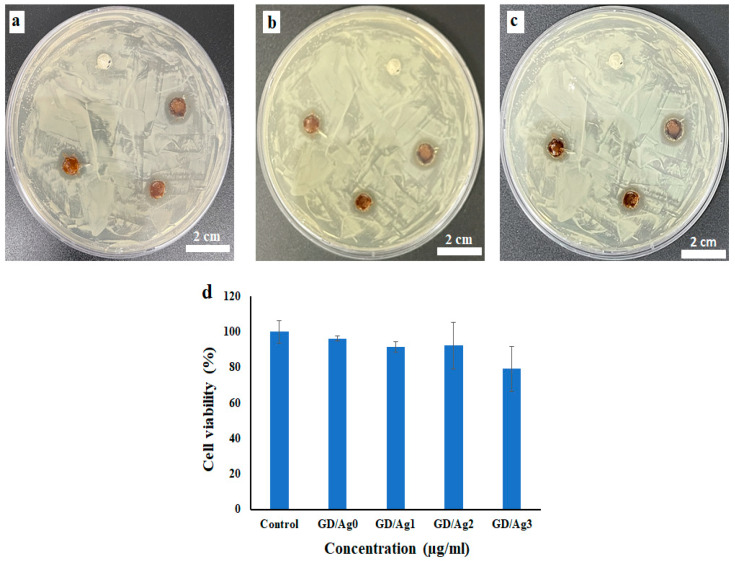
Photographs of the inhibition zones of Gel-Dex/Ag-NP nanocomposite hydrogels against *E. coli* in (**a**) 24 h, (**b**) 36 h, and (**c**) 48 h and (**d**) the in vitro cytocompatibility of nanocomposite hydrogels tested in HEK-293 cells.

The experiment using disk diffusion was conducted to study how effective Gel-Dex and its nanocomposite hydrogels with Ag-NPs are against *E. coli* (Gram-negative) bacteria. By visually observing the inhibition zones around the samples, the impact on bacterial growth was documented and included in Table 1 for reference. The results, illustrated in Figure 7a–c, indicate that the hydrogels with embedded Ag-NPs exhibited stronger antibacterial properties compared to the pure hydrogel, as shown by larger inhibition zones. The data in Table 1 highlight that the efficacy of the nanocomposite hydrogels is influenced by the concentration of Ag-NPs, irrespective of the bacterial strain used. Hydrogels containing higher amounts of Ag-NPs demonstrated superior antibacterial efficacy. The antibacterial mechanism of Gel-Dex/Ag-NP nanocomposite hydrogels is linked to the attachment of Ag-NPs to the bacterium’s cell wall, which leads to cell wall damage, protein leakage, disruption of intracellular components, and ultimately, cell death [50,51,52].

To evaluate the cytotoxicity of Gel-Dex/Ag-NP nanocomposite hydrogels on HEK-293 cells, the MTT assay was used. The cell viability was incubated with HEK-293 after 24 h with the results shown in Figure 7d. According to the Figure, the control group shows a baseline cell viability of approximately 100%. Cells treated with GD/Ag0 showed a slight decrease in viability compared to the control, maintaining around 96% viability. Increasing concentrations of GD/Ag (from GD/Ag1 to GD/Ag3) show a slight decrease in cell viability to approximately 92% to 79%, indicating the acceptable biocompatibility of the composite hydrogels [53,54].

## 3. Conclusions

This study developed and characterized a novel Gel-Dex hydrogel system integrated with Ag-NPs for advanced wound healing applications. The hydrogels were synthesized through a D-A click reaction and cross-linking Gel-MI with Dex-FA. Comprehensive analyses via NMR, FT-IR, SEM, EDS, and rheological tests confirmed the effective formation of the hydrogel network and uniform incorporation of Ag-NPs. The inclusion of Ag-NPs notably improved the mechanical strength and stability of the hydrogels. The nanocomposite hydrogels demonstrated adjustable swelling behaviors responsive to wound exudate, ensuring a moist environment conducive to healing. Cytotoxicity tests indicated that the hydrogels were biocompatible and safe for applications in wound care. In vitro antibacterial assays revealed that the hydrogels exhibited significant, concentration-dependent antimicrobial activity, effectively preventing bacterial infections. Rheological evaluations further supported that Ag-NPs reinforced the structural integrity and mechanical resilience of the hydrogels, enhancing their suitability for application as wound dressings. Collectively, the hydrogels’ tunable properties, sustained antimicrobial efficacy, and ability to maintain a sterile, moist wound environment collectively contribute to accelerated wound healing. Future research will focus on optimizing the hydrogel formulations for specific types of wounds and performing in vivo studies to validate their therapeutic potential. Additionally, exploring their application in drug delivery systems could further enhance their clinical utility. This Ag-NP-loaded Gel-Dex hydrogel represents a promising advancement in the field of wound care, offering a potent, multifunctional dressing for improved patient outcomes.

## 4. Materials and Methods

### 4.1. Materials

Gelatin porcine skin (Type A 300 Bloom, MW. 100 kDa), 2-furoic acid, and N-hydroxysuccinimide (NHS, 98%) were procured from Sigma Aldrich (Seoul, Republic of Korea). 1-ethyl-3-(3-dimethylaminopropyl)-carbodiimide hydrochloride (EDC.HCl, 99%), citric acid, Sodium carbonate (NaCO_3_), Magnesium sulfate (MgSO_4_), and NaCl were provided from TCI (Tokyo, Japan). Other reagents used in the study were dextran (150,000 gmol^−1^), 6-maleimidohexanoic acid, 4-Dimethylaminopyridine (DMAP), and *N*,*N’*-Dicyclohexylcabodiimide (DCC), which were supplied by Thermoscientific (Massachusetts, WLM, USA). Dimethyl sulfoxide (DMSO) and dichloromethane (DCM) were purchased by Duksan. Distilled water was the only type of water used in the study.

### 4.2. Measurements

A JEOL NMR spectrometer (JNM ECZ-400) was used for nuclear magnetic resonance (NMR) analyses. A Fourier transform infrared spectrometer (CARY 640, Agilent) was used to obtain FT-IR spectra. Rigaku (Ultima IV) was used to record the X-ray diffraction pattern of the samples using Cu-Kα radiation within the scan range of 2θ from 5° to 80° and a scan rate of 1°/min. The UV–vis absorption spectra of hydrogels containing Ag-NPs were recorded on a JASCO/V-770 Model UV–vis spectrophotometer. The morphology of hydrogels was investigated using a MIRA3 TESCAN FE-SEM. Energy-dispersive X-ray spectroscopy (EDS) was used to investigate the dimensions of Gel-MI/Dex-FA@Ag-NPs in the matrix of nanocomposite hydrogels.

The cells were grown in DMEM (HEK-293), supplemented with 1% antibiotic (penicillin/streptomycin, *v*/*v*) and 10% fetal bovine serum, and incubated at 37 °C in an environment that was humidified with 5% CO_2_.

### 4.3. Formulating Hydrogels by Preparing Precursors

#### 4.3.1. Synthesis of 6-Maleimidohexanoic Acid-Modified Gelatin (Gel-MI)

A Gel solution was created by dissolving the polymer in a mixed solvent system. A small amount of Gel was added to a vial containing a 5:4 ratio of water and DMSO. This was heated to 40 °C to dissolve the Gel fully. In a separate step, to a solution of 6-maleimidohexanoic acid (50 mg, 0.300 mmol) in DCM (1 mL) was added EDC.HCl (67.4 mg, 0.35 mmol) and NHS (39.3 mg, 0.34 mmol). The reaction mixture was stirred for 12 h, and white precipitate was observed. The solution was washed three times with 10% aqueous citric acid (1 mL each time), then three times with saturated aqueous NaCO_3_ (1 mL each time), and finally, saturated aqueous NaCl (1 mL). The organic extracts were dried over MgSO_4_, filtered, and concentrated under reduced pressure to afford 6-maleimidohexanoic acid *N*-hydroxysuccinimide ester as a white solid (54.6 mg) [55]. 6-maleimidohexanoic acid *N*-hydroxysuccinimide ester (0.125 g, 0.47 mmol) was weighed out and dissolved in 1 mL of DMSO. This solution was then incorporated into the Gel mixture and allowed to react over an extended period of time at an elevated temperature. After stirring at 40 °C for 24 h, the resulting light-pink solution was diluted with 10 mL of DI water and purified using dialysis membrane (MWCO 12–14 KDa) in DI water at 40 °C for 5 d with daily solvent exchange. Samples were aliquoted into 5 mL batches, frozen and lyophilized, and stored at −20 °C until further use [29].

#### 4.3.2. Synthesis of 2-Furoic Acid-Modified Dextran (Dex-FA)

Separately, 1.008 g of 2-Furoic acid (9 mmol) was activated by reacting it with 1.85 g of DCC (9 mmol) in DMSO (15 mL). Both reaction mixtures were stirred magnetically at room temperature for 3 h. The Dex-DMAP and FA-DCC solutions were then combined and stirred for a further 48 h. The resulting derivatized Dex product was precipitated by adding it to ice-cold acetone. The precipitate was isolated by centrifugation and dialysis against deionized water (MWCO 12–14 KDa). The pure Dex-FA conjugate was finally obtained through lyophilization for 72 h. This synthesis route is outlined in Scheme 2b [28].

#### 4.3.3. Formation of Nanocomposite Hydrogels Containing Ag-NPs

The process of cross-linking a hydrogel using the D-A reaction is demonstrated. First, different amounts of AgNO_3_ (0.005, 0.01, and 0.03 M) were dispersed in 100 µL of Gel-MI solution and the mixture was sonicated to remove any air bubbles. Afterward, the Dex-FA was gradually added to 100 µL of distilled water and stirred until the solution became homogeneous. In the next step, the solutions of Gel-MI/Ag-NPs and Dex-FA were then mixed and vortexed for 20 s to encourage the formation of a uniform phase. Subsequently, the reaction was carried out at 65 °C for 48 h to produce the nanocomposite hydrogel. The resulting nanocomposite hydrogel was subjected to freeze-drying for characterization using FT-IR and FE-SEM, as well as for determining the swelling ratio and pH-responsiveness. This simplified the process of hydrogel fabrication, as illustrated in Scheme 2c. To prepare a pure hydrogel without Ag-NPs, the same procedure could be followed. In this document, Gel-Dex/Ag-NP nanocomposite hydrogels are referred to as GD/Ag0, GD/Ag1, GD/Ag2, and GD/Ag3, depending on their Ag-NP content (0.0, 0.005, 0.01, and 0.03 M of AgNO_3_ content, respectively).

### 4.4. Rheological Analysis

The mechanical properties of Gel/Dex-based hydrogels were assessed by using dynamic oscillation of two parallel plates to measure the storage (G′) and loss (G″) moduli. During the frequency sweep test, a constant strain of 1% was maintained while changing the angular frequency from 0 to 100 rad/s. On the other hand, the dynamic oscillatory strain amplitude sweep experiments involved applying strain within the range of 0.1 to 10,000%, with the angular frequency kept constant at 10 rad/s.

### 4.5. Swelling Determination of Nanocomposite Hydrogels

An essential property of an effective wound dressing is its capacity to absorb exudate from the injured site. The swelling behavior of the dried hydrogel material was examined using a common gravimetric methodology. Specifically, the hydrogels were placed in 10 mL solutions buffered to physiological pH level of 7.4. At various time intervals, the swollen hydrogels were gently dried with filter paper and then weighed to assess their uptake of the surrounding fluid over time. This allowed for the evaluation of the dried hydrogel’s aptitude to swell when exposed to wound drainage, a principal attribute for dressings employed in promoting healing. The swelling ratio (SR) was determined using Equation (1), where W_t_ stands for the weight of the swollen hydrogel and W_0_ represents the weight of the dried hydrogel.

SR (%) = (W_t_ − W_0_/W_t_) × 100 (1)

### 4.6. In Vitro Cell Viability Assay

This study aimed to test the cytotoxicity of various formulations using HEK-293 cells. The test materials included pure hydrogel and nanocomposite hydrogels. The first step was to culture HEK-293 cells in 48-well plates at a density of 10,000 cells per well for 24 h in complete growth media. This allowed for the cells to attach. Next, the cells were exposed to sterile preparations of samples at different loadings for 24 h. Nanocomposite hydrogel cytotoxicity was assessed by incubating UV-sterilized gels with cells. Afterwards, the media was removed and cells were washed with PBS. A WST proliferation assay was then used to quantify cell metabolic activity after reagent addition and incubation. Absorbance readings from the plate reader were utilized to compare metabolic activity between treated and untreated control cells. Finally, the percentage of viable cells relative to controls was derived using a specified calculation to determine the cytotoxicity of each tested material. The percent cell viability relative to control samples was calculated using Formula (2).

Cell viability (%) = Absorbance of sample/Absorbance of control × 100(2)

### 4.7. Antibacterial Properties

The antibacterial activity of the hydrogels containing Ag-NPs was evaluated against Escherichia coli using the disk diffusion method. Agar was boiled in distilled water to dissolve it, and then it was sterilized by autoclaving at 120 °C for 1 h before cooling to room temperature. Lawns of the test bacteria were swabbed uniformly onto the agar plates. The hydrogel samples were placed onto the inoculated agar surfaces. The plates were inverted and incubated at 37 °C for 24, 36, and 48 h. The zone of clearance surrounding the samples, termed the zone of inhibition, was measured to assess the antibacterial efficacy of the hydrogels against Gram-negative *E. coli*.

### 4.8. Statistical Analysis

All physiochemical investigations were conducted in triplicate, while cell culture assays were performed with four replicates. The data are presented as the mean ± standard deviation.

## Figures and Tables

**Table 1:** Zones of inhibition (mm) against the tested bacteria for Gel-Dex/Ag-NP nanocomposite hydrogels.

		GD/Ag0	GD/Ag1	GD/Ag2	GD/Ag3
**Antibacterial diameter** (**mm**)	24 h	0	13.28	13.07	15.29
	36 h	0	12.75	12.98	14.53
	48 h	0	11.69	12.55	14.04

## Data Availability

The data presented in this study are available upon request from the corresponding author.

## Supplementary Materials

The following supporting information can be downloaded at: https://www.mdpi.com/article/10.3390/gels10060408/s1, Figure S1: The UV–vis spectra of nanocomposite hydrogels containing Ag-NPs (GD/Ag0, GD/Ag1, GD/Ag2, GD/Ag3).
