# Supplementary material for: The Diels-Alder Cross-Linked Gelatin/Dextran Nanocomposite Hydrogels with Silver Nanoparticles for Wound Healing Applications: Synthesis, Characterization, and In Vitro Evaluation
Source: Gels. 2024 Jun 19;10(6):408. doi: 10.3390/gels10060408 (PMC11202739; doi:10.3390/gels10060408)
Supplement: Supplementary file 1 [file gels-10-00408-s001.zip › gels-3061149-supplementary.pdf]

Supplementary

The Diels-Alder Cross-linked Gelatin/Dextran Nanocomposite Hydrogels with Silver Nanoparticles for Wound Healing Applications: Synthesis, Characterization, and In Vitro Evaluation

Iman Gholamali [1], Sung-Han Jo [1], Won Han [1], Juhee Lim [1], Ali Rizwan [2], Sang-Hyug Park [1,3,*] and Kwon Taek Lim [4,*]

[1] Industry 4.0 Convergence Bionics Engineering, Pukyong National University, Busan 48513, Republic of Korea; imangholamali1212@pknu.ac.kr (I.G.); josunghan91@gmail.com (S.-H.J.); hanwon0427@naver.com (W.H.); hee9752@naver.com (J.L.)

[2] Department of Smart Green Technology Engineering, Pukyong National University, Busan 48513, Republic of Korea; arizwan92@outlook.com

[3] Major of Biomedical Engineering, Division of Smart Healthcare, College of Information Technology and Convergence, Pukyong National University, Busan 48513, Republic of Korea

[4] Institute of Display Semiconductor Technology, Pukyong National University, Busan 48513, Republic of Korea

* Correspondence: shpark1@pknu.ac.kr (S.-H.P.); ktlim@pknu.ac.kr (K.T.L.); Tel.: +82-51-629-6406 (K.T.L.)

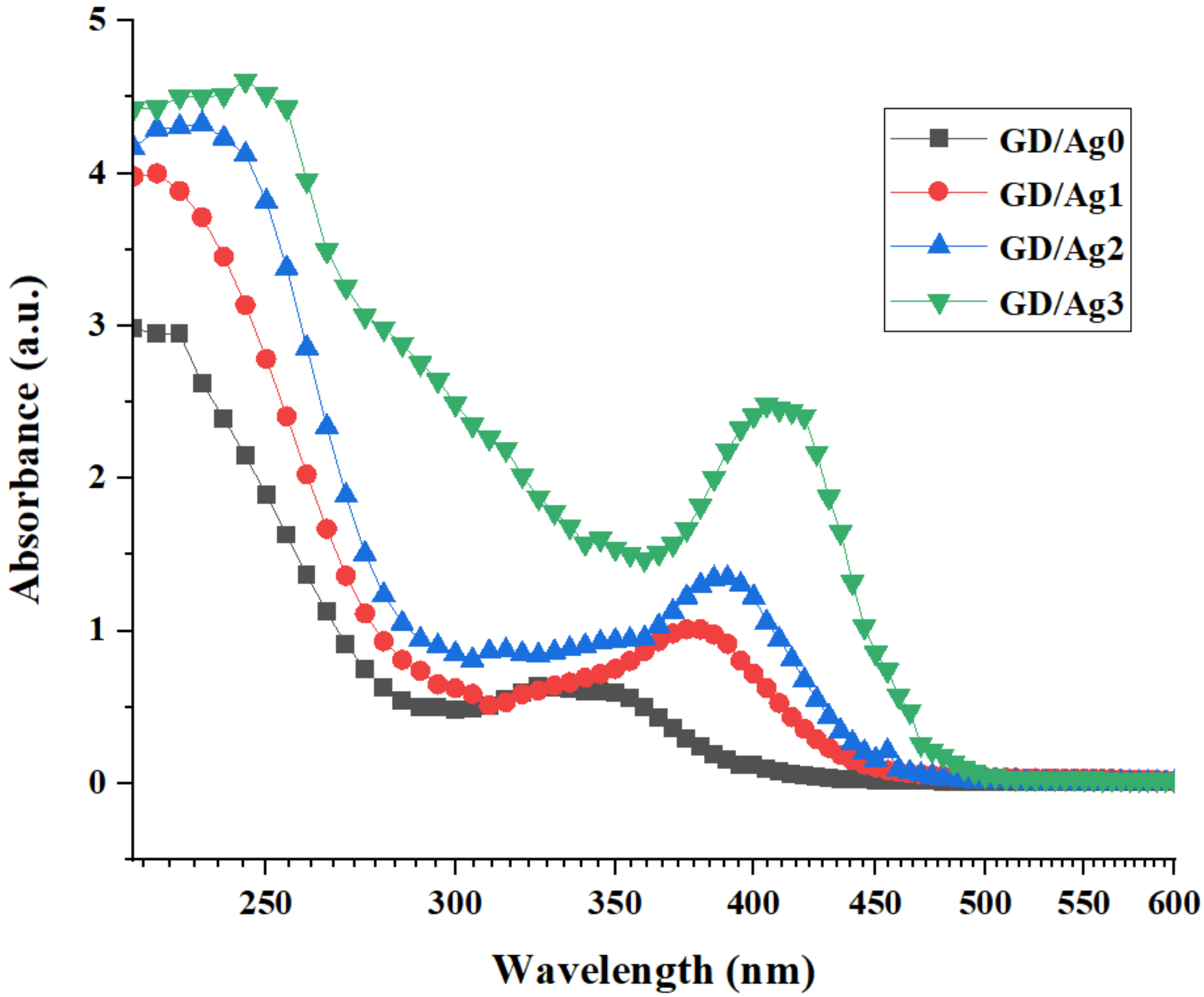

Figure S1. The UV–vis spectra of nanocomposite hydrogels containing Ag-NPs (GD/Ag0, GD/Ag1, GD/Ag2, GD/Ag3)
